# Rapidly Progressed Primary Intestinal Follicular Lymphoma with Elevation of Soluble Interleukin-2 Receptor Levels

**DOI:** 10.1155/2014/549248

**Published:** 2014-04-30

**Authors:** Masaya Iwamuro, Ryuta Takenaka, Atsushi Mori, Shigeatsu Fujiki, Takayoshi Miyake, Shoji Asakura, Hiroyuki Okada, Katsuyoshi Takata, Tadashi Yoshino, Kazuhide Yamamoto

**Affiliations:** ^1^Department of Gastroenterology and Hepatology, Okayama University Graduate School of Medicine, Dentistry, and Pharmaceutical Sciences, Okayama 700-8558, Japan; ^2^Department of Internal Medicine, Tsuyama Chuo Hospital, Tsuyama 708-0841, Japan; ^3^Department of Pathology, Tsuyama Chuo Hospital, Tsuyama 708-0841, Japan; ^4^Department of Internal Medicine, Okayama Medical Center, Okayama 701-1192, Japan; ^5^Department of Endoscopy, Okayama University Hospital, Okayama 700-8558, Japan; ^6^Department of Pathology, Okayama University Graduate School of Medicine, Dentistry, and Pharmaceutical Sciences, Okayama 700-8558, Japan

## Abstract

A 62-year-old Japanese male was diagnosed with primary intestinal follicular lymphoma involving the duodenum, jejunum, and rectum without lymph node involvement. The patient was classified as low risk by the follicular lymphoma international prognostic index (FLIPI) system. Treatment was deferred because he had no symptoms. Eleven months after the diagnosis, his soluble interleukin-2 receptor (sIL-2R) levels had risen from 383 to 617 U/mL. Lymphoma progression involving an enlarged perigastric lymph node was also documented. This report illustrates a case of rapidly progressed intestinal follicular lymphoma, suggesting the possible usefulness of sIL-2R levels as an indicator of lymphoma progression.

## 1. Introduction

Primary intestinal follicular lymphoma is a distinct subcategory of follicular lymphomas that was established within the last decade [1]. A wide variety of treatment options are available for patients with primary intestinal follicular lymphoma, including watch-and-wait, single-agent alkylating therapy, radiation therapy, systemic chemotherapy, and rituximab with various possible combinations [2]. Although there is insufficient evidence to identify the best or appropriate treatment, the watch-and-wait policy is considered an acceptable approach for intestinal follicular lymphomas, because in follicular lymphomas of nodal origin, delayed therapy has been reported to be associated with outcomes similar to those observed in treated patients [3–5]. In addition, due to the infrequency of this disease, the natural clinical course of primary intestinal follicular lymphomas has not been fully revealed.

We recently treated a patient with primary intestinal follicular lymphoma involving the duodenum, jejunum, and rectum. The patient presented without lymph node involvement, but progression with perigastric lymph node enlargement was documented 11 months after the initial diagnosis. The clinical course and strategies to follow-up untreated patients with this disease are discussed.

## 2. Case Presentation

**Figure 1:**
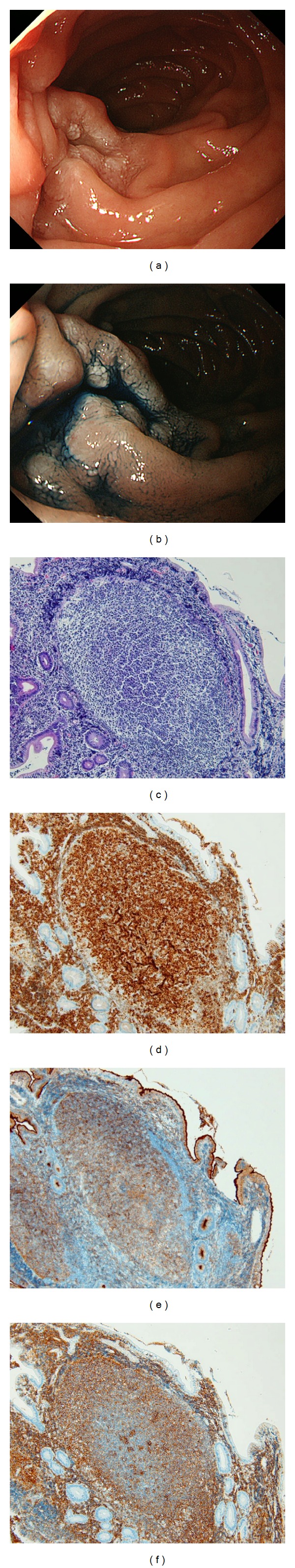
Esophagogastroduodenoscopy revealed whitish mucosa in the duodenum before (a) and after spraying indigo carmine dye (b). Biopsy specimens contained small- to medium-sized lymphoma cells forming lymphoid follicles ((c), hematoxylin and eosin stain). Those cells were positive for CD20 (d), CD10 (e), and BCL2 (f) but negative for CD3. Consequently, the diagnosis of follicular lymphoma was made.

A 62-year-old Japanese male underwent a curative resection of early gastric cancer by endoscopic submucosal dissection at the Tsuyama Chuo Hospital. The pathological diagnosis of the resected specimen was well-differentiated adenocarcinoma (34 mm dia.) located in the antrum, in which the carcinoma component was confined to the mucosal layer without lymphatic or vascular invasion. Esophagogastroduodenoscopy performed two months after the resection revealed whitish mucosa around the ampulla of Vater in the second portion of the duodenum (Figures 1(a) and 1(b)). Biopsy specimens were taken from the whitish villi. Pathologically, small- to medium-sized lymphoma cells forming lymphoid follicles were seen (Figure 1(c)). The cells were positive for CD20 (Figure 1(d)), CD10 (Figure 1(e)), and BCL2 (Figure 1(f)), but negative for CD3. The World Health Organization classification of the lymphoma cells was grade 1.

**Figure 2:**
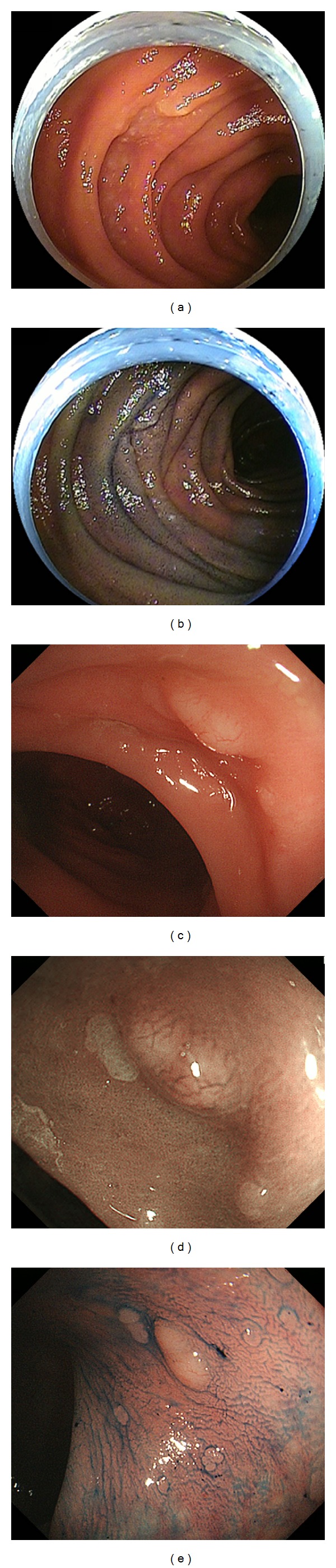
Double-balloon enteroscopy identified whitish granular areas in the proximal jejunum (a, b). Polypoid lesions with superficial telangiectasias were found in the rectum by colonoscopy (c–e). These lesions were pathologically confirmed as follicular lymphoma as well.

**Figure 3:**
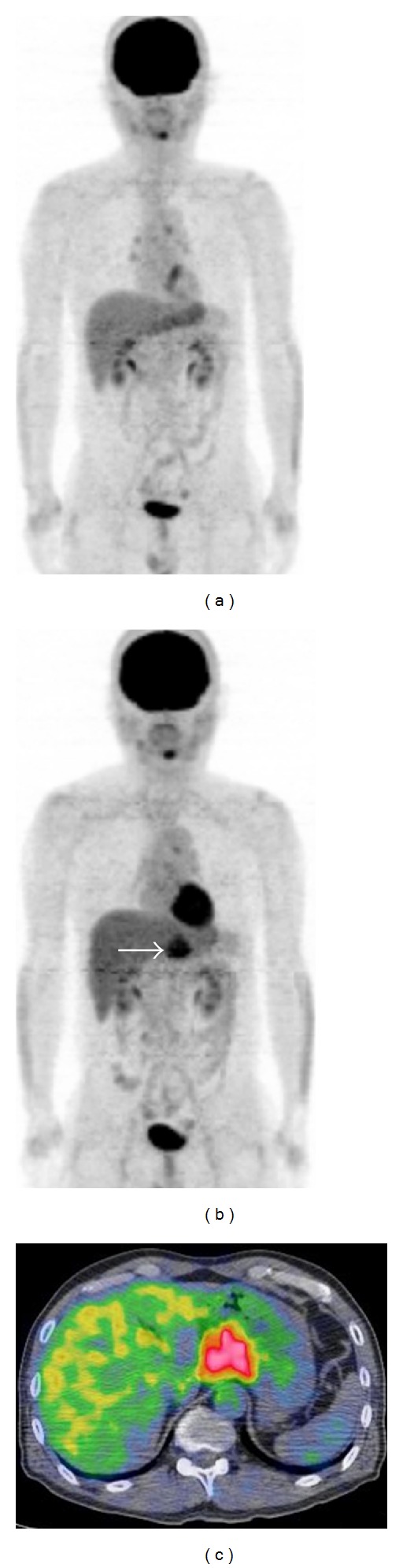
At the initial workup, a PET scan detected no positive tracer uptake in the whole body (a). However, an enlarged perigastric lymph node appeared 11 months after the initial diagnosis. The enlarged lymph node was positive for tracer uptake in the PET scan (b, c).

The patient had impaired glucose tolerance, but he had been taking no medication. A physical examination revealed no abnormalities, and there was no evidence of hepatosplenomegaly or peripheral lymphadenopathy. All laboratory findings, including the levels of lactate dehydrogenase (LDH) and soluble interleukin-2 receptor (sIL-2R), were within the normal ranges. Transoral and transanal double-balloon enteroscopy revealed two whitish granular areas in the proximal jejunum (Figures 2(a) and 2(b)). In colonoscopy, polypoid lesions with superficial telangiectasias were identified in the rectum (Figures 2(c)–2(e)). Biopsied specimens taken from the jejunal and rectal lesions also demonstrated the presence of lymphoma cells. Computed tomography (CT) scans of the neck, chest, abdomen, and pelvis showed neither lymphadenopathy nor a thickened gastrointestinal wall including the duodenum, jejunum, and rectum. An 18F-fluorodeoxyglucose positron emission tomography (PET) scan detected no positive tracer uptake in the whole body (Figure 3(a)). Bone marrow aspiration and biopsy revealed no lymphoma infiltration. Consequently, the patient was diagnosed with primary intestinal follicular lymphoma involving the duodenum, jejunum, and rectum. The clinical stage was considered stage I, based on the Lugano staging system for the classification of gastrointestinal tract lymphoma [6, 7]. The follicular lymphoma international prognostic index (FLIPI) score was 1, and thus the patient was classified as low risk by the FLIPI system [8].

The patient was assigned to a watch-and-wait policy because the lymphoma lesions were localized in the intestinal tract without lymph node involvement, and he had no symptoms. Eleven months after the diagnosis, the patient's sIL-2R levels had risen from 383 to 617 U/mL (upper limit: 530 U/mL) and a CT scan revealed an enlarged lymph node around the stomach. A PET scan detected positive tracer accumulation in the enlarged lymph node (Figures 3(b) and 3(c)).

**Figure 4:**
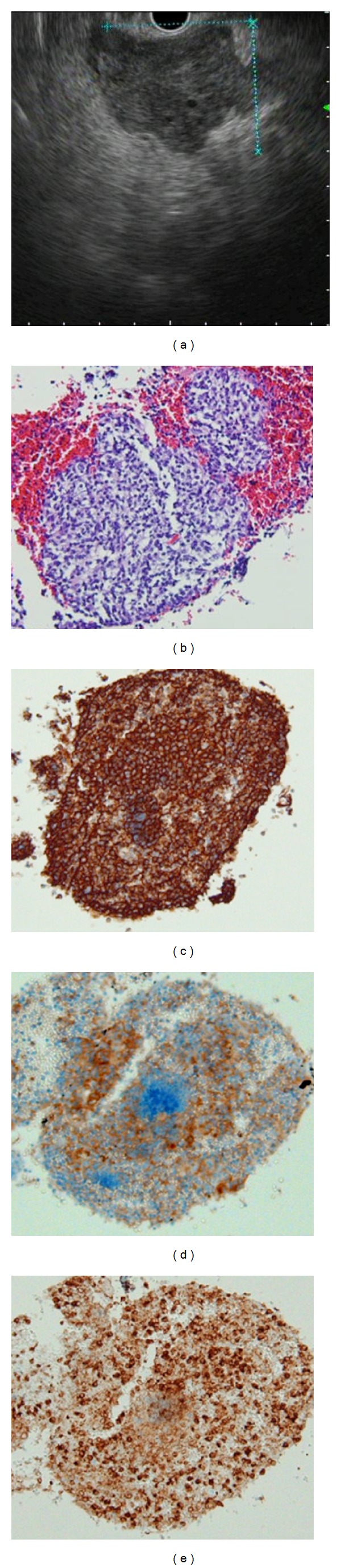
Endoscopic ultrasonography demonstrated an enlarged perigastric lymph node (a). Endoscopic ultrasonography-guided fine-needle aspiration biopsy revealed lymphoma cell infiltration in the lymph node (b) with positive staining for CD20 (c), CD10 (d), and BCL2 (e), indicating progression of the follicular lymphoma.

To determine whether the swelling of the lymph node was caused by follicular lymphoma involvement or gastric cancer recurrence, we performed an endoscopic ultrasonography-guided fine-needle aspiration biopsy (Figure 4(a)). The specimen obtained had lymphoma cells with positive staining for CD20, CD10, and BCL2 (Figures 4(b)–4(e)). The progression of follicular lymphoma, rather than a recurrence of gastric cancer, was thus pathologically confirmed. Rituximab monotherapy was initiated for the patient, and a partial response was achieved. A CT scan performed after the completion of the therapy showed that the swelling of the lymph node had reduced.

## 3. Discussion

In the present patient, although follicular lymphoma lesions were localized in the intestinal tract at the initial workup, progression with lymph node enlargement was observed 11 months after the initial diagnosis. Generally, follicular lymphomas are characterized by the nature of slow progression and even occasional spontaneous regression [9]. Despite the indolent nature, progression within 12 months after the initial workup is sometimes encountered in limited-stage (Ann Arbor stage I or II) follicular lymphomas of nodal origin. For example, Soubeyran et al. prospectively analyzed 26 patients who were assigned to a watch-and-wait strategy after achieving complete remission by the initial lymph node biopsy (stage I_0_) [10]. Among them, relapse within 12 months was observed in five patients (19.2%).

A prospective observational study of stage I follicular lymphoma enrolled in the U.S. National LymphoCare database revealed that 35/206 patients (17.0%) were deferred initial therapy, and progression within 12 months occurred in approximately 10% of these patients [4]. In a retrospective study of limited-stage follicular lymphoma patients by Michallet et al., progression within 12 months was observed in approximately 20% with a watch-and-wait policy [3]. Therefore, 10%–20% of limited-stage follicular lymphoma patients experience lymphoma progression within 12 months after the initial diagnosis.

Compared with the follicular lymphomas of nodal origin, only a few data are available regarding the natural history of primary intestinal follicular lymphomas because of the low prevalence of this disease entity. We assume that the primary intestinal follicular lymphomas have prognoses similar to those of limited-stage nodal follicular lymphomas, or they may have even better prognoses, especially in cases with duodenal involvement [11, 12]. Schmatz et al. reported that 2 of 24 previously untreated patients had developed nodal disease approximately 5 years after diagnosis [13]. A case series described by Misdraji et al. included five patients with stage I intestinal follicular lymphomas who were assigned to a watch-and-wait strategy [14]. Two of the five patients developed nodal disease 2 years and 4 years after diagnosis, respectively. Therefore, progression within 12 months, as described in the present case report, is considered infrequent in stage I intestinal follicular lymphoma patients.

A standardized examination schedule during the follow-up period for intestinal follicular lymphomas has not yet been established. For nodal cases, the European Society for Medical Oncology (ESMO) guidelines recommend history and physical examination every 3 months, blood count and routine chemistry every 6 months, and minimal adequate radiological or ultrasound examinations every 6 months for the initial 2 years [15]. We feel that it is better to follow up intestinal follicular lymphoma patients according to the schedules provided by the ESMO guidelines. However, radiological/ultrasound examinations at 6 months may be waived for primary intestinal follicular lymphomas (i.e., Lugano system staging I or II_1_), because progression within 12 months seems to be infrequent as noted above.

However, esophagogastroduodenoscopy should be performed to monitor duodenal involvement. Possible current topics to debate related to the endoscopic examinations include (i) what is the appropriate interval for esophagogastroduodenoscopy? (ii) should colonoscopy and enteroscopy be repeated during the follow-up period? and (iii) is a biopsy required at each endoscopic examination to screen for transformation? Further studies must be conducted to answer these questions.

It was noteworthy that our patient's sIL-2R levels were elevated when the lymphoma progression occurred. We recently analyzed sIL-2R levels at the initial diagnosis in 44 patients presenting with follicular lymphoma lesions in the gastrointestinal tract [16]. Patients with elevated sIL-2R levels are likely to have systemic involvement (Ann Arbor system staging IIIES/IV or Lugano system staging II_2_/IV), the involvement of five or more nodal areas, and bulky tumors in the gastrointestinal tract. Consequently, high sIL-2R levels reflect a large tumor bulk. Based on the results, we speculate that the elevation of sIL-2R levels during the follow-up period may be suggestive of relapse or progression in gastrointestinal follicular lymphoma patients. For nodal follicular lymphomas, Yoshizato et al. demonstrated that sIL-2R levels were increased after the relapse or regrowth of lymphoma lesions [17]. sIL-2R levels may thus be a good indicator for monitoring disease relapse or progression in intestinal follicular lymphoma patients as well. The presented patient is a proof-of-concept case of this issue.

In conclusion, we treated a patient with primary intestinal follicular lymphoma who experienced progression 11 months after the initial diagnosis. His sIL-2R levels were elevated when the progression occurred, suggesting the importance of sIL-2R levels as a tumor marker for intestinal follicular lymphomas.

## References

[1] Harris NL, Nathwani BN, Swerdlow SH, Swerdlow SH, Campo E, Harris NL (2008). Follicular lymphoma. *WHO Classification of Tumours of Haematopoietic and Lymphoid Tissues*.

[2] Yamamoto S, Nakase H, Yamashita K (2010). Gastrointestinal follicular lymphoma: review of the literature. *Journal of Gastroenterology*.

[3] Michallet AS, Lebras LL, Bauwens DD (2013). Early stage follicular lymphoma: what is the clinical impact of the first-line treatment strategy?. *Journal of Hematology & Oncology *.

[4] Friedberg JW, Byrtek M, Link BK (2012). Effectiveness of first-line management strategies for stage I follicular lymphoma: analysis of the National LymphoCare Study. *Journal of Clinical Oncology*.

[5] Advani R, Rosenberg SA, Horning SJ (2004). Stage I and II follicular non-Hodgkin’s lymphoma: long-term follow-up of no initial therapy. *Journal of Clinical Oncology*.

[6] Zucca E, Roggero E, Bertoni F, Cavalli F (1997). Primary extranodal non-Hodgkin’s lymphomas—part 1: gastrointestinal, cutaneous and genitourinary lymphomas. *Annals of Oncology*.

[7] Rohatiner A, d'Amore F, Coiffier B (1994). Report on a workshop convened to discuss the pathological and staging classifications of gastrointestinal tract lymphoma. *Annals of Oncology*.

[8] Solal-Céligny P, Roy P, Colombat P (2004). Follicular lymphoma international prognostic index. *Blood*.

[9] Freedman A (2012). Follicular lymphoma: 2012 update on diagnosis and management. *American Journal of Hematology*.

[10] Soubeyran P, Eghbali H, Trojani M, Bonichon F, Richaud P, Hœrni B (1996). Is there any place for a wait-and-see policy in stage I0 follicular lymphoma? A study of 43 consecutive patients in a single center. *Annals of Oncology*.

[11] Takata K, Okada H, Ohmiya N (2011). Primary gastrointestinal follicular lymphoma involving the duodenal second portion is a distinct entity: a multicenter, retrospective analysis in Japan. *Cancer Science*.

[12] Takata K, Sato Y, Nakamura N (2009). Duodenal and nodal follicular lymphomas are distinct: the former lacks activation-induced cytidine deaminase and follicular dendritic cells despite ongoing somatic hypermutations. *Modern Pathology*.

[13] Schmatz A-I, Streubel B, Kretschmer-Chott E (2011). Primary follicular lymphoma of the duodenum is a distinct mucosal/submucosal variant of follicular lymphoma: a retrospective study of 63 cases. *Journal of Clinical Oncology*.

[14] Misdraji J, Harris NL, Hasserjian RP, Lauwers GY, Ferry JA (2011). Primary follicular lymphoma of the gastrointestinal tract. *American Journal of Surgical Pathology*.

[15] Dreyling M (2010). Newly diagnosed and relapsed follicular lymphoma: ESMO clinical practice guidelines for diagnosis, treatment and follow-up. *Annals of Oncology*.

[16] Iwamuro M, Shinagawa K, Okada H, Takata K, Yoshino T, Yamamoto K (2014). Elevated soluble IL-2 receptor levels correlate with tumor bulk of follicular lymphomas with intestinal involvement. *Clinical Biochemistry*.

[17] Yoshizato T, Nannya Y, Imai Y, Ichikawa M, Kurokawa M (2013). Clinical significance of serum-soluble interleukin-2 receptor in patients with follicular lymphoma. *Clinical Lymphoma Myeloma and Leukemia*.

